# Supplementary material for: The formation of the light-sensing compartment of cone photoreceptors coincides with a transcriptional switch
Source: eLife. 2017 Nov 6;6:e31437. doi: 10.7554/eLife.31437 (PMC5685475; doi:10.7554/eLife.31437)
Supplement: Supplementary file 1. — List of genes defined as cone-specific (based on a cell type transcriptome study [Siegert et al., 2012]), ordered by fold change. (b) Transcription factors that switch off. List of transcription factors that switch off (log2 fold change >1), ordered by fold change. Only genes expressed above threshold (Materials and methods) are shown. (c) Mean percent change of switch pathways. Mean change in percent of all significantly up- or downregulated pathways. Pathways are ordered by p-value (at 1 million iterations). [file elife-31437-supp1.docx]

**Supplementary File 1a**

| **Gene symbol** | **Fold change (log_2_)** | **Expression level (log_2_)** |
| --- | --- | --- |
| *Opn1mw* | 9.1 | 10.5 |
| *Gckr* | 3.7 | 7.4 |
| *Agr2* | 3.5 | 6.1 |
| *Kcne2* | 2.9 | 8.7 |
| *Mogat1* | 2.7 | 2.2 |
| *Arr3* | 2.6 | 9.6 |
| *Slc25a35* | 2.6 | 3.8 |
| *Igj* | 2.5 | 5.4 |
| *Clca3* | 2.3 | 4.6 |
| *Pde6c* | 2.1 | 9.4 |
| *Uap1* | 1.8 | 6.0 |
| *Hspb6* | 1.8 | 8.4 |
| *Lcn2* | 1.7 | 2.3 |
| *Ppm1j* | 1.6 | 5.0 |
| *Pde6h* | 1.4 | 11.5 |
| *Osgep* | 1.4 | 6.4 |
| *Mpp6* | 1.3 | 7.4 |
| *Cckbr* | 1.2 | 6.6 |
| *Plekhb1* | 1.2 | 9.5 |
| *Opn1sw* | 1.1 | 10.9 |
| *Pde6d* | 1.0 | 7.1 |
| *Gnat2* | 1.0 | 10.0 |
| *Adrb1* | 1.0 | 5.5 |
| *Jam3* | 0.9 | 6.3 |
| *Acbd6* | 0.9 | 6.0 |
| *Rbp3* | 0.8 | 10.5 |
| *Tcta* | 0.7 | 5.4 |
| *Clpb* | 0.6 | 4.0 |
| *Slc1a2* | 0.6 | 8.0 |
| *Osbpl3* | 0.6 | 5.2 |
| *Krt18* | 0.5 | 7.2 |
| *Ppa2* | 0.4 | 6.7 |
| *Gngt2* | 0.3 | 9.4 |
| *Abcb4* | 0.2 | 1.8 |
| *Pcdh15* | 0.1 | 6.7 |
| *Otop3* | 0.1 | 6.8 |
| *Gulo* | 0.0 | 6.8 |
| *Cngb3* | -0.3 | 7.0 |
| *Ppp1r14d* | -0.4 | 2.3 |
| *Rxrg* | -0.7 | 5.1 |
| *Fabp7* | -1.9 | 8.9 |

**Supplementary File 1b**

| **Gene symbol** | **Fold change (log_2_)** | **Expression level (log_2_)** |
| --- | --- | --- |
| *Ascl1* | -7.6 | 32.3 |
| *Pou4f2* | -5.9 | 16.8 |
| *Sox11* | -5.2 | 22.3 |
| *Foxm1* | -5.1 | 3.9 |
| *Ebf3* | -5.1 | 50.9 |
| *Nr2e1* | -4.8 | 5.1 |
| *Tbx3* | -4.8 | 4.0 |
| *Ebf1* | -4.6 | 7.7 |
| *Zfp36l1* | -4.6 | 14.2 |
| *Sox8* | -4.4 | 8.5 |
| *Neurog2* | -4.4 | 4.6 |
| *Gli3* | -4.3 | 3.7 |
| *Myb* | -4.3 | 15.8 |
| *Hif3a* | -4.1 | 5.7 |
| *Pou4f1* | -4.0 | 14.0 |
| *Elavl2* | -3.9 | 6.0 |
| *Hmx1* | -3.9 | 11.0 |
| *Neurod4* | -3.7 | 116.7 |
| *Sox2* | -3.7 | 3.8 |
| *Insm2* | -3.7 | 12.0 |
| *Irx5* | -3.7 | 3.4 |
| *Lhx2* | -3.7 | 16.1 |
| *Myt1l* | -3.6 | 4.0 |
| *Satb2* | -3.5 | 7.8 |
| *Hes5* | -3.4 | 11.2 |
| *Notch1* | -3.3 | 19.2 |
| *Pax6* | -3.2 | 14.9 |
| *Sox4* | -3.2 | 13.9 |
| *Nfib* | -3.1 | 7.1 |
| *Scrt1* | -3.1 | 4.3 |
| *Arnt2* | -3.0 | 7.0 |
| *Nhlh2* | -3.0 | 3.0 |
| *Nrarp* | -2.9 | 5.1 |
| *Sox9* | -2.7 | 3.9 |
| *Tcf19* | -2.6 | 8.6 |
| *Klf13* | -2.5 | 4.6 |
| *Tbx2* | -2.4 | 10.5 |
| *Plagl1* | -2.3 | 6.0 |
| *Tfap4* | -2.3 | 3.9 |
| *Basp1* | -2.3 | 58.4 |
| *Klf7* | -2.3 | 18.7 |
| *Rab15* | -2.3 | 7.2 |
| *Zic2* | -2.3 | 4.7 |
| *Rem2* | -2.2 | 14.8 |
| *Zfp41* | -2.2 | 22.3 |
| *Nptxr* | -2.2 | 5.5 |
| *Mybl1* | -2.1 | 12.9 |
| *Carhsp1* | -2.1 | 15.2 |
| *Hes6* | -2.1 | 41.9 |
| *Tle3* | -2.1 | 15.7 |
| *Zfp423* | -2.1 | 17.8 |
| *Ebf4* | -2.1 | 6.9 |
| *Mcm6* | -1.9 | 5.9 |
| *Trip13* | -1.9 | 2.8 |
| *Onecut1* | -1.8 | 7.2 |
| *Cbx2* | -1.7 | 7.6 |
| *Prdm1* | -1.7 | 211.2 |
| *Fah* | -1.7 | 2.8 |
| *Ssbp3* | -1.7 | 59.9 |
| *Nfia* | -1.6 | 3.1 |
| *Six3* | -1.5 | 47.9 |
| *Hmgb2* | -1.5 | 20.5 |
| *Arid5b* | -1.5 | 6.7 |
| *Ldb1* | -1.5 | 43.9 |
| *Hes1* | -1.5 | 4.1 |
| *Isl2* | -1.5 | 10.1 |
| *Camk4* | -1.5 | 2.8 |
| *Lmo2* | -1.4 | 4.8 |
| *Fmnl2* | -1.4 | 19.7 |
| *Cdk4* | -1.4 | 68.1 |
| *Glis2* | -1.3 | 14.2 |
| *Caskin1* | -1.3 | 8.9 |
| *Tcf7* | -1.3 | 5.4 |
| *Zfp57* | -1.3 | 17.5 |
| *Otx2* | -1.3 | 533.6 |
| *Dach1* | -1.3 | 14.3 |
| *Mcm7* | -1.3 | 14.4 |
| *Hic2* | -1.2 | 4.7 |
| *Thra* | -1.2 | 35.9 |
| *Zfp189* | -1.2 | 4.4 |
| *Hmgcs1* | -1.2 | 55.6 |
| *Pax7* | -1.2 | 3.8 |
| *Rbl1* | -1.2 | 4.3 |
| *Satb1* | -1.2 | 24.2 |
| *Ski* | -1.1 | 18.3 |
| *Tle1* | -1.1 | 17.2 |
| *Sap30* | -1.1 | 10.7 |
| *Lsm11* | -1.1 | 5.7 |
| *Srebf2* | -1.1 | 20.5 |
| *Lmo4* | -1.1 | 11.4 |
| *Nfix* | -1.1 | 3.7 |
| *Pawr* | -1.1 | 4.0 |
| *Isl1* | -1.0 | 6.4 |
| *Tead1* | -1.0 | 20.5 |
| *Tcf3* | -1.0 | 12.6 |
| *Hmgb3* | -1.0 | 34.8 |
| *Tfdp2* | -1.0 | 9.2 |

**Supplementary File 1c**

| **Pathway** | **Mean change in %** |
| --- | --- |
| Metabolic pathways | 0.8 |
| Axon guidance | -50.8 |
| Phototransduction | 248.6 |
| Oxidative phosphorylation | 17.2 |
| p53 signaling pathway | -44.8 |
| Huntington's disease | 6.7 |
| Parkinson's disease | 7.6 |
| Cell cycle | -29.3 |
| Fructose and mannose metabolism | 31.8 |
| Rheumatoid arthritis | 29.7 |
| Colorectal cancer | -34.1 |
| Pathways in cancer | -23.5 |
| Regulation of actin cytoskeleton | -25.9 |
| Protein processing in endoplasmic reticulum | 2.3 |
| Retinol metabolism | 83.4 |
| Progesterone-mediated oocyte maturation | -30.3 |
| Glycolysis / Gluconeogenesis | 19.2 |
| Basal cell carcinoma | -41.4 |
| Olfactory transduction | 35.4 |
| Viral myocarditis | -41.5 |
| Hedgehog signaling pathway | -38.8 |
| Melanoma | -33.3 |
| Glycosaminoglycan biosynthesis - keratan sulfate | 43.6 |
| Ribosome | -25.3 |
| Pyruvate metabolism | 17.7 |
| Galactose metabolism | 23.3 |
| Glycerophospholipid metabolism | 7.6 |
| Amino sugar and nucleotide sugar metabolism | 9.8 |
| Lysosome | 1.5 |
| Focal adhesion | -23.6 |
| Alzheimer's disease | -0.5 |
| Notch signaling pathway | -29.3 |
| Adherens junction | -27.4 |
| Hypertrophic cardiomyopathy (HCM) | -30.1 |
| Citrate cycle (TCA cycle) | 13.4 |
| MAPK signaling pathway | -20.0 |
| Proximal tubule bicarbonate reclamation | 30.0 |
| Leukocyte transendothelial migration | -26.6 |
| TGF-beta signaling pathway | -27.2 |
| Dilated cardiomyopathy | -27.4 |
| Pancreatic cancer | -25.5 |
| Endometrial cancer | -27.2 |
| Arachidonic acid metabolism | 14.6 |
| Thyroid cancer | -31.8 |
| Arrhythmogenic right ventricular cardiomyopathy (ARVC) | -27.1 |
| Drug metabolism - cytochrome P450 | -37.7 |
| Glycerolipid metabolism | 5.7 |
| Nitrogen metabolism | 22.8 |
| Phosphatidylinositol signaling system | 1.5 |
| Chronic myeloid leukemia | -23.1 |
